# Quantitative Measurement of Rigidity in Parkinson’s Disease: A Systematic Review

**DOI:** 10.3390/s20030880

**Published:** 2020-02-06

**Authors:** María del Rosario Ferreira-Sánchez, Marcos Moreno-Verdú, Roberto Cano-de-la-Cuerda

**Affiliations:** 1 Department of Radiology, Rehabilitation and Physiotherapy, Universidad Complutense de Madrid (UCM), 28040 Madrid, Spain; marcom04@ucm.es; 2 Asociación Parkinson Madrid, 28014 Madrid, Spain; 3 Department of Physical Therapy, Occupational Therapy, Physical Medicine and Rehabilitation, Universidad Rey Juan Carlos (URJC), Alcorcón, 28922 Madrid, Spain; roberto.cano@urjc.es

**Keywords:** rigidity, assessment, Parkinson’s disease

## Abstract

Rigidity is one of the cardinal symptoms of Parkinson’s disease (PD). Present in up 89% of cases, it is typically assessed with clinical scales. However, these instruments show limitations due to their subjectivity and poor intra- and inter-rater reliability. To compile all of the objective quantitative methods used to assess rigidity in PD and to study their validity and reliability, a systematic review was conducted using the Web of Science, PubMed, and Scopus databases. Studies from January 1975 to June 2019 were included, all of which were written in English. The Strengthening the Reporting of observational studies in Epidemiology Statement (STROBE) checklist for observational studies was used to assess the methodological rigor of the included studies. Thirty-six studies were included. Rigidity was quantitatively assessed in three ways, using servomotors, inertial sensors, and biomechanical and neurophysiological study of muscles. All methods showed good validity and reliability, good correlation with clinical scales, and were useful for detecting rigidity and studying its evolution. People with PD exhibit higher values in terms of objective muscle stiffness than healthy controls. Rigidity depends on the angular velocity and articular amplitude of the mobilization applied. There are objective, valid, and reliable methods that can be used to quantitatively assess rigidity in people with PD.

## 1. Introduction

Rigidity is one of the primary symptoms of Parkinson’s disease (PD) [1], present in up to 89% of subjects [2]. The decrease in the levels of dopamine in the basal ganglia is found to be strongly associated with akinesia and rigidity [3]. However, diverse opinions exist as to the underlying pathophysiological mechanisms, especially regarding the contribution of each one to the final manifestation of the clinical signs. Some authors identify the main abnormalities as the excessive monosynaptic stretch reflexes, long-latency stretch reflexes, and the development of a tonic stretch reflex and a shortening reaction [4], while others highlight the influence of a reduced frequency in the discharge of neurons from the subthalamic nucleus and the alteration of the connectivity between the networks that link the cerebellum, the motor cortexes, and temporal, occipital, and caudate nuclei in patients with moderate PD [3].

The objective and standardized clinical evaluation of the motor signs of PD, especially for rigidity, is based on a semi-quantitative scoring system through clinical scales such as the Unified Parkinson’s disease Rating Scale (UPDRS) [5]. However, even a neurologist with expertise in movement disorders can commit an error of up to 20% in the accuracy of the diagnosis [6]. In recent decades, objective methods have been developed to quantify muscle tone. These methods are potentially useful for assessing rigidity in PD. One of these methods involves using servomotors, which mobilize a body segment at a desired speed. When this speed is constant throughout the range of motion (ROM) available, this is known as isokinetic dynamometry. This technique allows for the collection of information relative to the offered resistance as an objective measure of rigidity [7]. The application of these systems had been limited to the exploration of limbs (arm, wrist, knee, etc.) due to the difficulty of applying it to the trunk and hip [8]. However, in recent years its use has been extended to the axial musculature, motivated by the impact of stiffness at this level on gait, balance, and motor control [9].

Technologies such as portable sensors, which include inertial measuring units (IMUs) with accelerometers, gyroscopes, and magnetometers or potentiometers, offer systems that are non-invasive, trustworthy, quick, remote, economical, and objective [6]. Although they are not especially up-to-date (they were first used in this population in the 1990s) [6], their use has been recently included and extended in research and clinical environments for the evaluation of subjects with PD [10].

Another outstanding tool for the objective assessment of rigidity is electromyography, which allows for the detection of alterations in muscular tone and the definition of the underlying physiological mechanisms. Particularly characteristic in PD is the study of the cycle of recuperation of the H reflex, the recurrent inhibition, the F wave, or the polysynaptic skin reflexes, the values of which allow the identification of the presence of an alteration of the tone and the related modality [11].

Some of these technology-based methods that evaluate rigidity can also be used for other cardinal symptoms of PD, for example using wearable sensors, which have been used to analyze rest tremors [12,13,14], bradykinesia [15,16], and postural instability [17]. Nevertheless, these signs are relatively easy to assess objectively in comparison with rigidity, as rigidity is influenced by the position of the subject, the muscle assessed, or the modality of the examination. Hence, objective methods for assessment of the cardinal symptoms of PD are crucial for obtaining quantitative, reliable, and repeatable data.

In spite of the great advances made to date, the objective evaluation of rigidity continues to pose a challenge, and few studies have explored the reliability of the new evaluation devices in depth [10]. Therefore, the aim of this systematic review is to compile all quantitative methods for rigidity assessment in people with PD. This paper will focus on analyzing the validity and reliability of these methods in terms of muscle stiffness.

## 2. Materials and Methods

A systematic review of the literature was conducted following the Preferred Reporting Items for Systematic reviews and Meta-Analyses (PRISMA) statement [18].

### 2.1. Search Methods

We systematically searched the Web of Science, PubMed, and Scopus databases from January 1975 to June 2019. Key words used were “rigidity”, “hypertonia”, and “muscle stiffness”, crossed with “evaluation”, “assessment”, “quantification”, “Parkinson”, and “parkinsonian”, utilizing pertinent Boolean terms and specific search filters from each database. Detailed search strategies are shown in Table 1. Two authors independently searched data and analyzed abstracts and titles to decide if a study met the eligibility criteria. In the case of disagreement, this was solved by a third independent author. We only included studies published in English, and abstracts and protocols were excluded.

### 2.2. Data Extraction and Analysis

A systematic review was conducted by two independent authors. We used a standardized data extraction protocol, collecting information about population, intervention, methods, results, and outcome measures. For each study we defined the design, sample size, joint explored, type of system used for the quantification of rigidity, evaluation protocol, rigidity outcome measures, and other clinical variables related to PD severity, functional independence, quality of life, and results. Finally, both authors reached an agreement about each extracted data item. Studies employing objective assessment methods of muscle rigidity in PD patients were selected.

### 2.3. Assessment of the Methodological Rigor of the Included Studies

Due to the high methodological heterogeneity of the included studies, we could not use a standardized tool to assess the risk of bias. Thus, we used the Strengthening the Reporting of observational studies in Epidemiology Statement (STROBE) checklist for all types of observational studies (case–control, cohort, cross-sectional) as a method to assess methodological rigor [19]. This scale contains a list of 35 items that evaluate the degree, rigor, and amount of information provided in each article section. Each item is scored by “yes”, “no”, or “unclear”, so that “yes” is equivalent to 1 point and “no” or “unclear” are equivalent to 0 points. Thus, high scores indicate greater degrees of confidence or reliability of information provided by the authors. Six checklist items (Table 5, columns 12c, 13b, 14b, 14c, 16b, and 16c) were not applicable to any of the included studies. These items gathered information related to processing of missing data, how loss of follow-up was addressed in cohort studies (none was included in this review), gave information about the reasons for non-participation at each stage, and presented results with relative and absolute risk values. For this reason, scores were turned into a 29-point scale instead of the original 35-point scale.

## 3. Results

### 3.1. Description of the Selection Process

**Figure 1:**
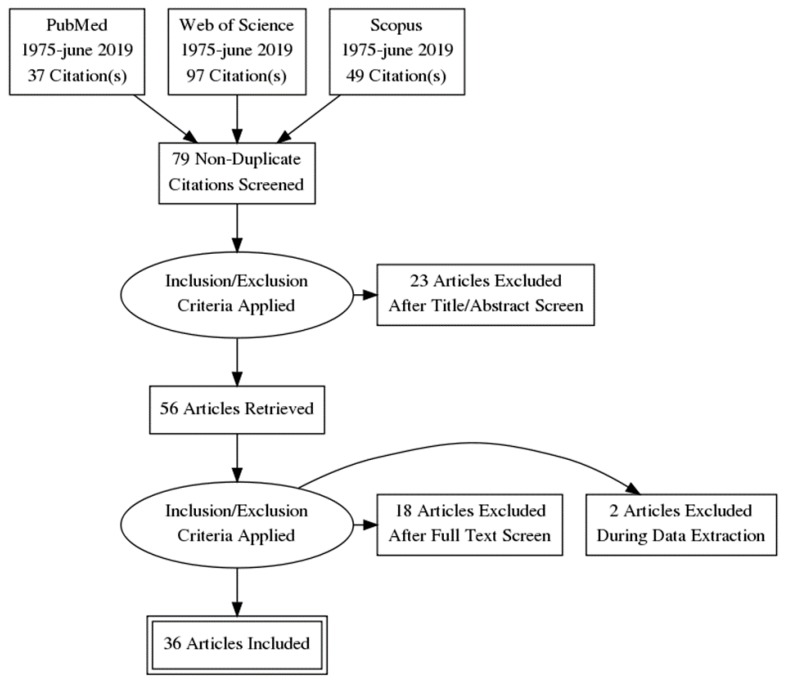
Flow diagram.

A description of the selection process is described in Figure 1. Initial searches generated a total of 2535 results, of which 167 were potential studies. After removing duplicates and applying eligibility criteria, 36 studies were finally included.

### 3.2. Description of the Included Studies

#### 3.2.1. Methods Evaluating the Mechanical or Neurophysiological Properties of the Muscle

Eleven studies were included, which are summarized in Table 2. Of these, five used electromyography [20,21,22,23,24], three used myotonometry [25,26,27], and three used elastography [28,29,30]. The study design chosen in all cases was case–control, except for the study by Hayashi et al., who used a pre–post design. The sample size was quite low for all the studies (27.64 ± 18.92, with a range of 11–77; mean ± SD). Five out of 11 studies did not reach 20 participants, whilst only 3 of 11 papers had a sample size ≥30 participants.

Electromyography (EMG) has typically been the method of choice for studying the muscular physiology of patients with PD, showing a good correlation with clinical scales that evaluate rigidity [21]. The examination protocol used varies radically between authors. While some examine the muscles at rest or in submaximum contraction [20], others do so by analyzing the response to passive stretch-shortening cycles, imposed by an examiner [21,22,23,24]. In addition, the cycles can be carried out with constant or variable angular velocity and the joint amplitude varies between 20° and 90°.

Various authors have found that the musculature of people with PD has higher EMG activity than that of healthy controls, both at rest [20,23] and during stretching and subsequent relaxation [22]. The most representative muscular group of general rigidity is still the subject of debate. For example, Solopova et al. [23] found levels up to 1.7 times higher in stiffness (measured in N m/(m^2^ deg)) in hamstrings in people with PD, and also in the tibialis anterior and gastrocnemius lateralis muscles (up to 1.3 times). They also found that in people with PD, the plantar flexors were significantly stiffer than the dorsal flexors, and that this stiffness did not respond to the administration of levodopa [23]. On the other hand, other authors have analyzed other muscular groups that are both larger (biceps and triceps brachii) [22] and smaller (hand muscles) [20], but the specific correlations of a single muscular group with the clinical rigidity scales have not been analyzed.

EMG analysis allows one to obtain relevant data regarding stiffness in PD. For example, it has been shown that after a submaximal contraction maintained over 16 s, people with PD need between 15–20 min to relax the muscles to levels close to EMG silence, while healthy people are able to achieve this almost instantaneously [20]. It has also been found that subjects with PD have greater EMG activity in the middle and final phases of a muscular stretch (moments of maximum muscular tension), but not in the previous phases (moments of low tissue stress) [22].

Myotonometry (also known as myometry) is another instrumental palpation method that has been used. With this method, natural oscillation data for a biological tissue is obtained, showing acceleration signals of that oscillation and simultaneously computing the “tension” parameters and the biomechanical and viscoelastic properties. This method offers data on muscle tone (Hz) and stiffness (N/m), among other information. It has been proven to be a tool with high reliability and reproducibility (Intraclass Correlation Coefficient, ICC > 0.9) that correlates well with the clinical rigidity measurements of the UPDRS [27]. Its use has been studied exclusively in the musculature of the arm (biceps and triceps), obtaining data both at rest and in slightly maintained contraction, in both the belly of the muscle and the tendon [25]. It has always been used to study the most affected side, collecting an average of 20 consecutive records per second, from which the average stiffness value in a given muscle is obtained. Marusiak et al. [25] found that people with PD have higher rigidity values in comparison with healthy controls, and that this is related to changes in the viscoelastic properties of the muscle rather than neurophysiological changes, since electromyographic and mechanomyographic (MMG) data did not show differences between groups.

Ultrasound allows for the visualization of soft tissues such as muscle and the study of the related mechanical properties. Elastography was developed from this technique, which allows the stiffness of a biological tissue to be determined, the data for which correlates with the UPDRS rigidity subscale in patients with PD [28,29]. Through an ultrasound transducer placed in the muscular belly, gray-scale or color images are obtained, along with objective measures such as the so-called “longitudinal elastic modulus” (or Young’s modulus), which indicates the ability of the muscle to deform in the presence of an external compression. It has been shown that Young’s module correlates well with the motor part of the UPDRS, and the intra- and inter-observer reliability of the technique is good (ICC > 0.7). In relation to this measure of rigidity, differences have been found between people with PD and healthy controls, being significantly higher in the former than in the latter [28].

Other authors have used elastography to obtain the so-called “stress ratio” as the main parameter, which relates the average stress of the muscle tissue with the average reference stress (the higher the stress ratio, the greater the muscle stiffness). Brachial bicep muscle stress has been analyzed, using a 31% increase in the stress ratio as the optimal cut-off point for the diagnosis of PD, the sensitivity and specificity of which were 0.91 and 0.86, respectively. Inter- and intra-observer reliability was also good (ICC > 0.8). In addition, it has been found that the stress ratio is significantly higher in subjects with PD compared to healthy controls [29], and that this is a parameter that is modified with the administration of levodopa, both in subjects with PD and in subjects with parkinsonism [30]. However, improvements in the stress ratio after administration of L-dopa were significantly greater in the group with PD compared to the group with parkinsonism [30].

#### 3.2.2. Methods Using Sensors for the Capture and Analysis of Movement

Nine studies were included, which are summarized in Table 3. Of these, 5 examined the wrist [31,32,33,34,35], 2 examined the elbow [36,37], 1 examined the trunk [38], and 1 examined the entire body [39]. The evaluation protocol varied significantly between studies as the design and objectives were different. All studies used at least one sensor to capture the range of movement (gyroscope) and another for angular speed and acceleration (accelerometer). In addition, most included a potentiometer or magnetometer that obtained torque data.

The mean sample size was 28.11 ± 21.50 (mean ± SD) for these methods. These studies presented different designs, combining reliability studies and case–control designs. Reliability studies related with these technologies presented a lower sample size (<20 subjects), and they only include PD patients. In contrast, case–control studies had a bigger simple size (75% exceeded 50 participants).

Three studies used sensors to detect the rigidity of the muscles around the wrist in a clinical setting [31,32,33]. When comparing the data collected in people with PD and healthy controls, two of them concluded that the sensors are useful for detecting rigidity and that there is a correlation between their data and that provided by clinical measures [32,33]. One of the sensors was also able to detect which people with PD had rigidity or not, which correlated with traditional measures [32]. In addition, two concluded that their respective systems were able to differentiate the “on” and “off” states [31,32].

Two studies placed sensors on the wrists of the subjects in a surgical setting [34,35]. The subjects underwent deep brain stimulation surgery to compare the data obtained in this protocol and the accuracy of the sensors with the data obtained by a neurosurgeon. Both studies found that the sensors showed good correlation with the opinion of the clinician, were useful for detecting rigidity with high reliability, and their data were representative of the improvement obtained by the subjects after surgery.

To identify axial (trunk) rigidity, one study [38] analyzed the kinematics of pelvic and thoracic angular rotation during the gait of subjects. Through a Vicon-type system using accelerometers in both tibiae, a method of relating the coordination between trunk and pelvis rotation was developed, therefore generating an objective measure (so-called “relative phase” measure) that is comparable between subjects with and without PD. This work concluded that the average duration of the relative phase is significantly reduced in people with PD compared to healthy subjects. The variability was also significantly reduced, indicating greater axial rigidity in these subjects.

One study [36] showed that a sensor placed on the elbow correlated well with clinical measures and found that people with PD had significantly higher values than healthy controls (both young and old) in terms of average torque. Another sensor on the elbow showed that rigidity decreased significantly after administrating medication. In addition, this sensor showed that there were differences in the qualitative interpretation of the rigidity by the different examiners who took part in the study [37].

The main finding of the study that used a center of pressure (CoP) platform to establish a “postural stiffness measure” (k) through a mathematical model was finding positive correlations with the parameters of rigidity, bradykinesia, and posture in terms of UPDRS (among others). Thus, the usefulness of this model was demonstrated, which uses the information from the anteroposterior displacement of the CoP to obtain objective rigidity data [39].

#### 3.2.3. Electro-Mechanized Methods Adhered to the Body for Joint Mobilization

This type of system receives is referred to as a servomotor and consists of a motor mechanism that mobilizes the joint passively at the desired speed and amplitude while collecting quantitative information on the response to stretching in terms of position, torque, force, work, and angular displacement.

Eighteen studies were included, which are summarized in Table 4. Eight examined the wrist joint [40,41,42,43,44,45,46], 6 examined the elbow [47,48,49,50,51,52], and 1 examined thumb [47], knee [53], cervical spine [54], and trunk [55]. The case–control design was the most frequent design used, even though 18.75% used a case series design. In contrast to Section 3.2.1 and Section 3.2.2, these are the ones that presented the widest sample size variability, ranging from 8 to 127 subjects. The mean sample size was 38.75 ± 28.02 (mean ± SD). Additionally, 43.75% of the studies presented a sample size <30, whilst 25% exceed 50 participants.

Due to the anatomical and biomechanical differences of the evaluated regions, there is some variability between the protocols developed. However, they all follow a common action procedure according to the following guidelines. First, the subject’s exploration position was determined in all cases, allowing passive and isolated movement of the segment to be explored with the adjacent segments correctly stabilized. Subsequently, the joint movement to be studied was selected based on its respective kinematic parameters. The points to be defined included the articular range (ROM) in which the movement was to be executed, the speed (usually fast and a slow speeds were applied), the rest time between each test, and the number of repetitions to be performed.

Studies that evaluated the rigidity in the wrist [40,43,44,46] determined that the ideal range for the detection of rigidity is between 60° and 90°, with the wider ranges being more sensitive. Likewise, contradicting the classic definition of rigidity, it has been demonstrated through the included studies that rigidity, in addition to suffering variations along the articular range, is dependent on the speed at which the stretching is performed [40,42,44]. Speeds below 70°/s are not very sensitive when examining rigidity [40] and speeds above 300°/s produce a sharp increase in stretch reflexes causing saturation [47]. It was finally determined that the ideal speed for this evaluation is 140–190°/s [40]. Therefore, the greater the range of movement and the greater the speed of stretching, the greater the test sensitivity that is achieved. Additionally, in this range the correlation of these measurements with the clinical scales is increased.

In studies examining the elbow, the ROM of exploration varies between 40° and 70° [47,48,49,50,51,52], and similar data is obtained with respect to the influence of speed on the torque compared with in wrist studies [49]. All studies on the elbow reveal that the torque, working values per unit of movement, and stretching reflexes are significantly higher in people with PD compared to healthy controls [47,48,49,50,52].

No consensus has been reached about the possible relationship between the severity of PD and the stretch reflex due to the diversity of results obtained in these studies [41,45,47]. Likewise, there are large discrepancies in relation to the influence of dopaminergic medication on rigidity [45,46,50].

The study conducted on the interphalangeal joint of the thumb reinforces the relationship between stretch speed and the size of the long-latency reflex in subjects with severely affected PD [47].

The combination of the electro-mechanized system with electromyography allowed for significant differences to be found between the group of subjects with PD versus healthy controls in terms of the torque and electromyographic activity during the knee flexion and extension movement at different speeds [53]. In addition, this reinforces the hypothesis of the existence of a coactivation of the shortened musculature in addition to the elongated musculature during stretching, especially in the extension movement.

The study carried out on the cervical spine showed high peak torque values in the subjects with PD compared to the controls before stretching, as well as difficulty in relaxing the cervical musculature afterwards [54]. In line with the studies described above, the study performed on the trunk confirms that the greatest resistance is obtained at the end of the ROM in flexion and extension movements at different speeds, showing a good correlation between rigidity and other clinical variables [55].

### 3.3. Methodological Rigor of Included Studies

**Figure 2:**
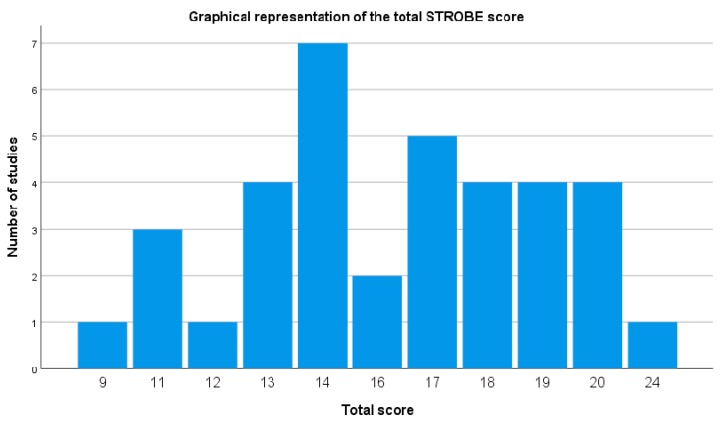
Graphical representation of the total STROBE score.

The STROBE checklist scores of the included studies are summarized in Table 5 and Figure 2, which describes the graphical representation of the total STROBE score. The mean score of included studies was 15.92 points, with a standard deviation of 3.33. The minimum score was 9 points and the maximum score was 24 points. Most of studies (>70%) obtained a positive rating in items regarding the title and abstract (Table 5, columns 1a and 1b), introduction (Table 5, column 2), study design (Table 5, column 4), definition of the variables of interest (Table 5, column 7), sources (Table 5, column 8) and quantification Table 5, column (11), description of statistical methods (Table 5, column 12a), data on sample characteristics (Table 5, column 14a), reporting of outcome measures (Table 5, column 15) and statistical estimates Table 5, column (16), and summary of key results with reference to study objectives (Table 5, column 18).

Most of the studies (>50%) obtained negative or unclear ratings for items regarding specific objective statements (Table 5, column 3), eligibility criteria and outcome measures (Table 5, column 6a), predictors and other definitions (Table 5, column 7), description of efforts to address potential sources of bias (Table 5, column 9), methods used to examine subgroups (Table 5, column 12b), methods used to match cases and controls (Table 5, column 12d) and to carry out sensitivity analysis (Table 5, column 12e), reports of numbers of individuals at each stage of study (Table 5, column 13a), discussion of limitations (Table 5, column 19) and of generalizability (external validity) of the study results (Table 5, column 21), and the source of funding (Table 5, column 22).

## 4. Discussion

This paper compiles the instrumentalized methods used to assess, quantify, and analyze the rigidity associated with PD. This systematic review focuses both on analyzing the reliability and objectivity of the methods, as well as on presenting the findings that compare muscle stiffness in subjects with PD and in healthy people with the different methods used in scientific literature.

Our results show that all the methods compiled in the present review have a good correlation with clinical scales for the quantification of rigidity (UPDRS subscale, clinical rigidity score). In addition, these methods demonstrate good test–retest reliability and the data does not appear to be influenced by the examiner’s experience, the moment of exploration, or the change of exploration. However, for servomotors, there are influences of both angular velocity and joint amplitude on correlations. At higher speed and greater amplitude, there is a better correlation with clinical scales, such as the clinical rigidity score (CRS) [40]. Other authors found correlations between the data obtained by servomotors and other clinical variables, such as the stage of evolution (Hoehn and Yahr scale) and the degree of independence in activities of daily living (Schwab and England scale) at both fast and slow speeds [55].

All the instruments reviewed offer the possibility of obtaining objective data in the presence of muscle stiffness compared to healthy subjects. Therefore, they could be considered useful tools in the diagnosis and evaluation of extrapyramidal rigidity and in examination of the response to different treatments.

An important part of the diagnostic analysis of a certain clinical condition is the direct comparison of the accuracy of the different methods used. In this sense, no comparative studies have been conducted analyzing the different instruments that have been compiled in this review. Therefore, although all of them have been proven to be valid, reliable, and sensitive. It would be interesting to analyze the diagnostic accuracy in terms of psychometric properties to decide which of these methods is the gold standard for identifying rigidity in PD. With the current data, the choice of method should be made by considering the experience the evaluator has with the technique, the ease of data processing, and the simplicity of the evaluation protocol.

There were no significant differences (*p* > 0.05) in sample size among the methods used for the assessment of rigidity, as we did not perform comparisons between muscle study methods and sensor methods (*p* = 0.882), muscle study methods and servomotors (*p* = 0.231), or sensor methods and servomotors (*p* = 0.335). The sample size was very varied among the included studies, ranging from 4 to 127 with a mean ± SD of 32.42 ± 24.20. Of the above studies, 55.55% had a sample size <30 subjects, whilst only 22.22% had a sample size greater than 50. This was the main limitation for these studies. Interpretation and generalization of the results should be performed with caution because of the small sample sizes.

### 4.1. Neurophysiological Mechanisms of Rigidity

The neurophysiological mechanisms underlying rigidity in PD are still a matter of debate. In global terms, there are currently two hypotheses that partially explain the physiopathology. One of them focuses on changes produced at the spinal level, mainly of the Ia and Ib interneurons, as a consequence of an altered input by the reticulospinal tract [56]. The high fusimotor activity from neuromuscular spindles has also been proposed as a contributor to this alteration [4].

In any case, the modification at the medullary level would produce changes that generate two parallel and partially related phenomena: tonic increase in the stretch reflex [4] and increase in the shortening reaction [57]. The shortening reaction is an abnormal response of a muscle that has been shortened and responds with a contraction to the change in length. This is a paradoxical response and has been observed prominently in people with PD in the “off” state using EMG data during a servomotor analysis [58].

The other theory that explains the rigidity focuses on the finding of an exacerbated long-latency reflex (M2) that appears in the EMG of people with PD [59,60]. Long-latency reflexes are virtually silent in the EMG of a healthy subject and are understood to be a supra-spinal response to stretching that is mediated by circuits located in the sensory motor cortex [61]. Through the modification of the input to the supplementary motor area, PD would produce an alteration of these reflexes, which would explain the active rigidity and difficulty of people suffering from this disease to relax their muscles at will [20,56,62]. These results are consistent with the reviewed studies that show that the long-latency stretch reflex correlates with the severity of rigidity [45,47] and responds positively to pallidotomy [24]. These data were found through evaluation with servomotors and EMG, respectively.

The degree of contribution of the neural and non-neural components to the final rigidity remains a matter of debate. The non-neural component corresponds to the alteration of the viscoelastic mechanical properties of muscle fibers and passive connective tissues [63]. The neural component is inherent in the pathophysiology of PD and is due to the previously mentioned alterations (long-latency reflexes, shortening reaction, etc.). It seems that the neural component is modifiable with both the administration of medication [42] and with deep brain stimulation in the subthalamic nucleus [22,24,34], while the non-neural component is not. Some studies suggest that the non-neural component contributes more to rigidity [26], others postulate that it is the neural [41], while others find an equivalent participation of both [42]. The inconsistency of these data can be explained by the different methodology used (type of instrument, examination protocol, information analysis algorithms) or the variability of the collected sample. In any case, the literature seems to suggest that there is a partial contribution of both components to total rigidity. This fact is supported by data obtained with both elastography [28,29] and myotonometry [26] or with servomotors [41,42].

### 4.2. Relevant Parameters in the Evaluation of Rigidity: Angular Speed, ROM, and Other Factors

Some of the tools reviewed allowed in-depth investigation of the biomechanical factors that influence rigidity. For example, using servomotors we can analyze the influence of the speed of mobilization on rigidity. Traditionally, it has been postulated that rigidity is not speed-dependent, which is the main difference with spasticity. However, the studies analyzed in this review contradict this premise [21,40,42,44,52]. For example, in the wrist, it has been found that the speeds between 140–190°/s are the ones that “trigger” the rigidity the most and are the most sensitive to detection [40]. Speeds below 70°/s are practically not able to differentiate between normal tone and rigidity [40], and speeds above 300°/s produce such a degree of stretching reflexes that causes saturation in both healthy subjects and those with PD. These speeds are also not useful for detecting rigidity (torque values are maximal in both groups) [47]. In fact, some authors have examined subjects with spasticity and rigidity and concluded that the servomotor, despite being able to differentiate between normal tone and hypertone, is not able to identify the type of hypertonia based on the response to speed. This implies that rigidity or spasticity discrimination in terms of dependence or independence of angular velocity is impossible with current instruments [49,52].

The joint amplitude used in the rigidity test also determines the results of the rigidity [21,40,42,44,46]. In the wrist, the ideal range for rigidity detection is between 60° and 90°, with the wider ranges being more sensitive. Several authors have shown that displacement in wider ranges (90° versus 60°) is associated with a greater increase in rigidity in terms of work and angular momentum [46]. In contrast to angular velocity, joint amplitude could be a discriminating element between spasticity and rigidity, since some servomotors show that spastic muscles have a progressively greater tension relative to ROM, while rigid ones have a higher tension but are constant throughout the entire stretching range [49]. This would involve two different patterns of response related to position, which can also be distinguished from that of healthy subjects.

Other factors such as the frequency of mobilization or the number of repetitions also influence rigidity. This must be taken into account in the clinical context, since some studies determine that there is a progressive decrease in torque throughout the repetitions performed [53,55].

### 4.3. Dopaminergic Medication and Rigidity

Some authors have studied the reliability of their instruments by examining whether they are able to detect the “off” vs. “on” change produced by the administration of levodopa and other antiparkinsonian drugs [30,31,42,43,46,50]. Theoretically, the rigidity responds well to medication [5,64], but frequently the outcome measures used (clinical scales such as UPDRS and others) in clinical trials seem to suffer from an obvious subjectivity, poor sensitivity to change, and variable inter- or intra-examiner reliability [5]. On the other hand, the use of instrumentalized methods could provide greater assurance regarding the efficacy of pharmacotherapy (and other treatments), since they offer quantitative information. With the current data, we can conclude that there are sensors [31] and servomotors [42,43,50] capable of detecting the “off” vs. “on” change in terms of objective muscle stiffness, while other instruments do not find statistically significant differences after the administration of medication in rigidity values [46]. More research is required through instrumentalized methods to know the degree of effectiveness of medication on rigidity.

### 4.4. Limitations

This work has several limitations. First of all, we could not use a standardized tool to assess risk of bias of included studies due to high methodological heterogeneity. Thus, we included studies with various types of methodological designs. Only studies published in English were included. Finally, included studies showed several intrinsic limitations, considering that most of them did not report data regarding the diagnostic accuracy (sensitivity, specificity, predictive values) of the instrument employed, or that the instruments have only been studied in some joints or muscle groups, thus limiting the comparison with other parts of the body and reducing extrapolation. The protocol diversity utilized by authors hampers direct comparisons between studies, restricting the generalization of the conclusions of the present review.

## 5. Conclusions

Devices used to quantitatively evaluate rigidity with objective data in PD patients are described in the literature, which used three different methods: servomotors, inertial sensors, and biomechanical and neurophysiological muscle measurement. All these methods show good validity and an adequate correlation with clinical instruments for muscle stiffness assessment. In addition, the most recently developed methods reviewed (myometry and elastography) have demonstrated excellent reliability properties, both for test–retest (myometry) and for inter- and intra-examiner reliability (elastography).

Compared with healthy subjects, people with PD show greater values of rigidity, both at rest and during passive mobilization. Their muscles exhibit an objective hypertonia in terms of electromyographic activity, response to biomechanical deformation, and stretch resistance, which can be detected with instrumentalized tools.

When external mobilization of a joint is used to assess rigidity severity, this protocol depends both on the angular velocity of the mobilization and on the articular amplitude. Therefore, depending on the mobilized joint, it can be important to determine these parameters when clinically evaluating this phenomenon.

## Figures and Tables

**Table 1:** Detailed search strategy.

Database	Specific Filters	Search Strategy and Key Words
Web of Science	Timespan: all years (1900–2019). Tag field: title. Document type: article.	[*“rigidity”* OR *“hypertonia”* OR *“muscle stiffness”*] AND [*“evaluation”* OR *“assessment”* OR *“quantification”*] AND [*“Parkinson”* OR *“parkinsonian”*]
PubMed	Publication dates: January 1975–June 2019. Article type: clinic study, evaluation study, observational study, validation study. Language: English.	
Scopus	Publication dates: January 1975–June 2019. Document type: article.	

**Table 2:** Methods evaluating the mechanical or neurophysiological properties of the muscle.

Study	Study Design	Sample	Assessment Method	Joint Explored	Evaluation Protocol	Results
Cantello, 1995 [20]	Case–control	8 people with PD 8 healthy controls	Surface EMG on hand muscles.	Hand	Tested on the most affected side. Basal muscle activity was measured (at rest) with a maintained contraction for 16 s. Number of action potentials by motor unit, firing rate, and recruitment order were obtained.	EMG activity at rest was significantly higher in PD in comparison with healthy controls after relaxation time. This was a consequence of the discharge of a greater number of different motor units (5.9 ± 2.7 in control group vs. 20.9 ± 5.3 in PD group) (excessive recruitment). Firing rate was not significantly different between groups.
Fung, 2000 [21]	Case–control	20 people with PD 10 healthy controls	Surface EMG on flexor carpi radialis and extensor carpi radialis while a servomotor passively moves the wrist.	Wrist	Tested in “off” phase, and on the most affected side. In controls, half of the tests performed on each side. Tested at rest and with contralateral activation maneuver. Five records in 60° of ROM with rest intervals were collected. Tests were performed at two cycle frequencies (at 1 and 1.5 Hz). A qualitative scale to evaluate rigidity (CRS) was administered.	Examiner tended to use quasi-sinusoidal mobilizations at frequencies between 0.5 and 2 Hz, and with articular amplitudes of ± 40°. Activation produced a significant increase in CRS when compared to rest in both groups, but the magnitude of this increase was greater in PD. Angular impulse scores were significantly higher in PD, both at rest and with activation maneuver. There were no differences between groups for work scores in any case. In PD, high correlation between CRS and angular impulse was confirmed (work score was not well correlated). Better correlations were found at 1.5 Hz. Activation maneuver increased angular impulse scores.
Levin, 2009 [22]	Case–control	13 people with PD 8 healthy controls	Surface EMG on biceps brachii and triceps brachii.	Elbow	Tested in “off” phase (deep brain stimulation -DBS- electrodes turned off) and “on” phase (30 s after DBS electrodes turned on). Tested on the most affected side. Elbow was flexo-extended (90° amplitude) by an examiner from total extension to 90° flexion (it was registered by a goniometer). Cycles were made at 0.5 Hz. All individuals were tested twice while EMG was recorded for 180 s.	People with PD had greater EMG activity only during middle and final stretch phases compared with healthy controls. During the first two phases, there were no significant differences between groups. There were different EMG patterns when comparing people with high scores (UPDRS 3–4) and lower scores (0–1) in clinical rigidity. In both muscles, people with PD with low clinical rigidity exhibited differences compared with healthy controls.
Solopova, 2014 [23]	Case–control	25 people with PD 22 healthy controls	Surface EMG (bilateral) on rectus femoris, biceps femoris, tibialis anterior, and gastrocnemius lateralis muscles. A potentiometer sensor fixed coaxially with lower limb joints.	Lower limb (hip, knee, and ankle)	Two recordings in “off” phase and one in “on” phase. Passive movement at 7°/s of each joint in isolation, in a range of ± 20° (ankle ± 10°). Each movement was examined three times. The resistance force, the shortening reaction (mean value of EMG values eliminating the basal activity in each test), and the latency period of the test were recorded.	Hip muscles stiffness was 1.5 and 1.7 times higher in people with PD than healthy controls for flexors and extensors, respectively (*p* < 0.05). In the hip, flexors exhibited greater rigidity than extensors (*p* < 0.05) in both groups, as well as in the knee (1.5 and 1.6 times). In the ankle, dorsiflexors were 1.3 times more rigid in people with PD than controls. Plantar flexors exhibited similar stiffness levels in both groups (*p* = 0.3). Plantar flexors were significantly more rigid than dorsal flexors in PD patients. Levodopa improved hip and knee stiffness but not ankle stiffness.
Hayashi, 2001 [24]	Pre–post design	11 people with PD	Surface EMG on flexor carpi radialis and extensor carpi radialis.	Wrist	Assessment before (“off” state) and 2–3 months after (“on” state) pallidotomy surgery. A servomotor moved the wrist while EMG is recorded and M1 and M2 and their amplitudes were obtained. The two conditions were passive (participant was instructed not to resist the movement) and active (participant was instructed to suddenly resist).	There were no significant differences between active and passive conditions at either preoperative or postoperative stages for M1 parameter or basal activity. For M2 parameter, significant differences were found after pallidotomy in active condition (M2 amplitude decreases). In passive condition, the tendency was a decrease, but statistical signification was not reached. Pallidotomy had no effects on inherent muscle stiffness.
Marusiak, 2018 [25]	Case–control	8 people with PD 10 healthy controls	Myotonometry (Myoton-3 device) on biceps brachii (short head) and triceps brachii (long head).	Elbow	Tested on the most affected side; in controls, on dominant side. Test at rest (20 consecutive recordings with 1 s interval between each) and with a voluntary contraction of 10% of the MVC. In the second condition, elbow was placed at 15° flexion and the subject had to hold a 2 kg weight for 1 min while 20 recordings were collected. Test–retest reliability was assessed.	Myotonometry had high test–retest reliability (ICC > 0.9), both at rest and at 10% of the MVC.
Marusiak, 2011 [26]	Case–control	12 people with PD 12 healthy controls	Myometry (Myoton-3 device) on biceps brachii and triceps brachii (belly and tendon).	Elbow	Tested in “on” state. Twenty consecutive recordings were collected in the following order (biceps belly, biceps tendon, triceps belly, triceps tendon). EMG and MMG data were also collected, as well as the elbow ROM at rest.	Myometry had excellent reproducibility. There were no differences between groups for EMG and MMG. There were differences between groups in the myotonometric examination of the bicep belly and tendons. In the triceps this was true in the tendons (*p* < 0.05), but not so in the belly of the triceps.
Marusiak, 2010 [27]	Case–control	8 people with PD 10 healthy controls	Myotonometry (Myoton-3 device) on biceps brachii (short head).	Elbow	Tested in “on” phase, and on the most affected side; in controls, on the dominant side. Twenty records were collected, in addition to EMG and MMG. Reproducibility, group differences, and correlation with clinical scales and EMG/MMG were analysed.	Good reproducibility in both groups (ICC > 0.9). PD group exhibited higher stiffness values than control group (203 ± 22 N/m vs. 192 ± 8 N/m, *p* = 0.004). There was a positive correlation between myotonometry and clinical rigidity scores.
Du, 2016 [28]	Case–control	46 people with PD 31 healthy controls	Shear wave elastography (AixPlorer) on biceps brachii.	Elbow	The transducer was placed in the muscle belly to collect grayscale images and then over a color code. Three measurements were collected and de Young’s modulus (longitudinal elasticity modulus) was obtained.	Young’s modulus had a moderate positive correlation with UPDRS-III (*r* = 0.646; *p* = 0.000). Inter- and intra-examiner reliability was good (ICC = 0.74 and 0.78, respectively). For Young’s modulus, there were differences between PD (54.94 ± 20.91 Kpa on the most affected side and 47.77 ± 24 Kpa on less affected side) and healthy people (24.44 ± 5.09 Kpa).
Gao, 2016 [29]	Case–control	14 people with PD 10 healthy controls	Elastography (Logic E9 ultrasound scanner) on biceps brachii	Elbow	Tested in “off” phase. A 5-s cycle was performed to collect the deformation-relaxation cycle. Three measurements were made on each subject. To exert the same pressure on the skin, a weight was placed on the end of the apparatus. Biceps stress, reference stress, and stress ratio (mean biceps stress divided by mean reference stress) were measured.	Stress ratio was significantly higher in the PD group with respect to control group (3.3 ± 0.27 vs. 2.65 ± 0.6; *p* = 0.00011). A correlation was found between stress ratio and UPDRS rigidity subscale score. Reliability of measurements was good (ICC intra-observer = 0.88, ICC inter-observer = 0.84).
Gao, 2016 [30]	Case–control	11 people with PD 7 persons with parkinsonism	Elastography (Logic E9 ultrasound scanner) on biceps brachii.	Elbow	Tested in “off” and “on” phases. A 5 s cycle was performed to collect the deformation–relaxation cycle. Three measurements were made on each subject. To exert the same pressure on the skin, a weight was placed on the end of the apparatus. Biceps stress, reference stress, and stress ratio (mean biceps stress divided by mean reference stress) were measured.	PD group exhibited significant differences in stress rate in “off” phase with respect to “on” phase (2.86 ± 0.51 vs. 4.06 ± 0.78; *p* = 0.02). This was also true for parkinsonism group (2.56 ± 0.23 vs. 2.87 ± 0.37; *p* = 0.14). There were significant statistically differences between groups for the increase in the stress ratio parameter (*p* = 0.0007) in favor of PD.

Abbreviations: CRS, clinical rigidity score; EMG, electromyography; MMG, mechanomyography; MVC, maximal voluntary contraction, PD, Parkinson’s disease; ROM, range of motion; UPDRS, unified Parkinson’s disease rating scale.

**Table 3:** Methods using sensors for the capture and analysis of the movement.

Study	Study Design	Sample	Assessment Method	Joint Explored	Evaluation Protocol	Results
Van den Noort, 2017 [31]	Technology reliability	4 people with PD	Sensor complex (PowerGlove system) with gyroscopes, accelerometers, and magnetometers.	Wrist and hand	Test in “on” and “off” phase. After calibration, the wrist was passively flexo-extended by an examiner while sensors collected data regarding angular velocity, ROM, torque, rigidity, impulse, and work.	There were significant differences between “off” and “on” phases. ROM increased, while torque, rigidity, impulse, and work scores decreased.
Caligiuri, 1994 [32]	Case–control	29 people with PD 25 healthy controls	A portable transducer with gyroscope and potentiometer.	Wrist	Test performed at rest and with contralateral activation maneuver. Examiner passively moved the wrist (flexion and extension) within a 45° ROM over at least 15 cycles.	The group of PD with clinical rigidity presented higher mean instrumental rigidity scores than control group (1.57 vs. 1.09; *p* < 0.0001), as well as higher scores than PD without clinical rigidity group (1.57 vs. 1.05; *p* = 0.0001). During “off”- vs. “on” phase examination (*n* = 4), instrumental rigidity score was reduced by 26% after levodopa/carbidopa administration.
Byung Kyu, 2011 [33]	Case–control	45 people with PD 12 healthy controls	A sensor with potentiometer, load cell, and accelerometer.	Wrist	Tested in “on” phase. Movement was applied by an examiner. Random flexion and extension (1–5 s) and random rest (2–10 s) within a ROM of −35° and +35°. Six flexion and extension cycles were applied, between 2–4 times each, bilaterally.	Damping viscosity constant was well correlated with clinical rigidity measured by UPDRS. Tissue resistance (viscosity) was greater during extension. Velocity dependence of rigidity was more pronounced in subjects with greater clinical rigidity scores. Viscosity measure was useful for detecting muscle stiffness.
Kwon, 2014 [34]	Technology reliability	8 people with PD	A sensor with potentiometer, load cell, and accelerometer.	Wrist	Tested in “off” (before DBS surgery) and “on” states (after surgery). “Off” and “on” data were compared with each other and with data collected by a neurologist.	Damping viscosity constant was well correlated with clinical rigidity (Spearman coefficient = 0.77), and was the variable that improved the most after DBS (2.38 pre vs. 0.39 post; *p* < 0.001). The other variables showed moderate to low correlation (Spearman < 0.7), although all improved significantly after surgery (*p* < 0.05) with the exception of mechanical impedance.
Costa, 2015 [35]	Technology reliability	10 people with PD	Sensor with a gyroscope, accelerometer, and magnetometer.	Wrist	ROM and angular velocity were obtained during flexion and extension while DBS surgery was performed. These data were compared with each other and with data collected by a neurologist.	The device was capable of distinguishing between rigidity and normal tone (83.9%), and its measurements were correlated with clinical rigidity with an error margin of 8.24% ± 7.95%. The system was useful for discriminating discrete rigidity levels and for detecting cog wheel rigidity with good sensitivity (0.93).
Ghika, 1993 [36]	Case–control	20 people with PD 15 healthy controls 20 healthy controls (young)	A semimanual device with goniometer and potentiometer.	Elbow	An examiner passively moved the elbow with 0.67 Hz angular velocity (using a metronome) in a ROM of 45°. The sensor recorded data on angular movement and torque.	Obtained values were correlated with clinical rigidity. PD group had significantly greater values compared with the others in the mean torque (right side 0.391 Nm/degree and left side 0.388 Nm/degree vs. 0.054 and 0.044 Nm/degree, respectively, for the age-matched controls; *p* < 0.05).
Prochazka, 1997 [37]	Proof of concept	14 people with PD	Sensors with potentiometer to measure the force applied by the examiner at the wrist. A sensor	Elbow	Wrist sensors measured the force applied by the examiner to move the forearm. Additionally, a string was placed with its limits adhered to the shoulder and the forearm of the subject. The string measured elbow ROM during the examination.	Rigidity was significantly reduced after medication administration. Clinical evaluation had very low reliability. The results of the evaluation were influenced by factors such as velocity or ROM, and also attention of the subject, reinforcement maneuver, and distractors. Impedance and rigidity profiles that were generated by examiners were similar, but high differences in the qualitative interpretation of rigidity existed.
			with a string to measure the elbow ROM.			
Van Emmerik, 1999 [38]	Case–control	27 people with PD 11 healthy controls	Optoelectronic system with infrared LEDs on pelvis and thorax. Accelerometers on both tibias.	Trunk	Analysis during gait. Angular rotation of pelvis and trunk and their velocities were obtained in order to record a relative phase measure along with its variability.	PD group exhibited lower relative phase mean duration and lower variability of this factor. in both groups, relative phase increased as velocity increased, without any group differences. There were no differences between groups for stride duration or its variability.
Lauk, 1999 [39]	Technology reliability	18 people with PD	Centre of pressure (CoP) platform (Kistler).	Whole body	Tested in “on” phase. Ten 30 s tests with open eyes were made. A 60 s rest period between tests was allowed. A “postural rigidity measure” (k) was obtained through a mathematical model that used information regarding AP displacements of the CoP. Reliability was assessed by randomly by separating the tests into 2 groups of 5 tests each and comparing them. UPDRS was administered.	Positive correlations with the UPDRS items of rigidity, bradykinesia, posture, lower limb agility, and retropulsion test were found. There was no correlation with rest tremor, postural tremor, or the getting up items. Results showed that the measure proposed had significant correlations with other relevant variables.

Abbreviations: AP, anteroposterior displacement; CoP, center of pressure; DBS, deep brain stimulation; ROM, range of motion; UPDRS, unified Parkinson’s disease rating scale.

**Table 4:** Electro-mechanized methods attached to the body for joint mobilization (servomotors).

Study	Study Design	Sample	Assessment Method	Joint Explored	Evaluation Protocol	Results
Teräväinen, 1989 [40]	Case–control	29 people with PD 12 healthy controls	Servomotor with position feedback.	Wrist	Movements of flexion and extension were applied in a passive way and in contralateral activation, over a range of angular velocities from 12°/s to 240°/s, and over angular displacements from 15° to 30°.	Higher velocities gave more sensitive results than the lower ones in the assessment of rigidity. Wide angular displacements and velocities gave the highest correlations between objective evaluation and the CRS.
Zetterberg, 2015 [41]	Case–control	25 people with PD 14 healthy controls	Servomotor (NeuroFlexor).	Wrist	Flexion and extension movements were applied in a range of 50°, at two velocities of 5°/s and 236°/s, under passive and dynamic conditions. Bilateral exploration: less affected side was dominant side; more affected side was non-dominant side.	PD showed greater resistance and neural component than controls, with no difference in components between sides. Total resistance was greater in the dynamic test in both groups.
Xia, 2016 [42]	Case–control	14 people with PD 14 healthy controls	Servomotor and surface EMG.	Wrist	Tested without antiparkinsonian medication (at least for 12 h) and under medication (45–60 min before the test). Flexion and extension movements in minimum displacements.	There were significant differences in neural components between “on” and “off” states.
Xia, 2009 [43]	Case series	12 people with PD	Servomotor with EMG.	Wrist	Tested with and without antiparkinsonian medication. Flexion and extension movements were applied in a range of motion of ±30° at two velocities: 50°/s and 280°/s.	There was a correlation between the torque resistance and the ration values of EMG. There was a correlation between the activation of the stretched and shortened muscles. Direction and speed of the passive movement had a great influence on rigidity.
Zito, 2018 [44]	Case–control	4 people with PD 18 healthy controls	Servomotor (exoskeleton wrist resistance robot (WRR)).	Wrist	Flexion and extension movements in a range of motion from −60° to +30° were applied, at 10°/s and 50°/s. The most affected wrist was evaluated in PD group and non-dominant wrist was evaluated in controls.	Significative differences were found in position, speed. and torque for both groups, at every speed.
Perera, 2019 [45]	Case–control	8 people with PD 8 age-matched healthy controls 8 young healthy controls	Servomotor (Bionics Institute Rigidity Device (BiRD)).	Wrist	A cycle per second of flexion/extension movement was executed. Each evaluation consisted of 15 cycles.	Force rate was significantly greater in PD vs. controls, both at rest and during activity. This allowed for detection of “on” and “off” states. A moderate congruence (R = 0.68) was shown with Movement Disorders Society-Unified Parkinson’s Disease Rating Scale (MDS-UPDRS).
Powell, 2012 [46]	Case series	18 people with PD	Servomotor and EMG.	Wrist	Tested with and without antiparkinsonian medication. Flexion and extension movements in a range of 60° and 90°, at two speeds of 50°/s and 280°/s.	Higher displacements (90 vs. 60) were associated with greater rigidity. There were no significant differences between “on” and “off” states.
Rothwell, 1983 [47]	Case–control	47 people with PD 12 healthy controls	Servomotor and EMG.	Elbow and thumb	Thumb: movement of the interphalangeal joint at about 10° of flexion. Elbow: resting position at 90° flexion. Forces of 8, 16, and 24 N were applied.	Stretch reflexes were augmented in patients with moderate PD. The was a positive correlation between the severity of the disease and the magnitude of the stretch reflex. The saturation of the reflexes occurred at velocities greater than 300°/s in both patients and controls.
Kirollos, 1996 [48]	Case–control	2 young healthy controls 2 elderly healthy controls 2 elderly with activation phenomenon but not hypertonia at rest or other parkinsonian signs 2 untreated PD people and mild–moderate rigidity	Servomotor.	Elbow	Contralateral activation by squeezing a sphygmomanometer cuff. Test was started with an acclimatization phase for 2 min to the passive arm movement. Then, 6 tests were executed at 10 s intervals in both resting and contralateral grasping states.	PD required higher work values by unit of displacement.
Lee, 2002 [49]	Case–control	16 people with PD 12 hemiparetic spastic people 12 healthy controls	Servomotor with sensors and electromyography of biceps and triceps.	Elbow	Flexion and extension movements in a range of motion of 75°. Stretch velocities were set at 40, 80, 120, and 160°/s, were applied randomly for	Hemiparesis and PD patients reported significantly greater torque values at higher speeds. Healthy controls revealed low values.
					PD patients, and a single speed of 175°/s was selected for healthy controls.	
Relja, 1996 [50]	Case–control	24 people with PD 103 healthy controls	Tonometer with a transductor device.	Elbow	Basal and activated rigidity were tested with and without antiparkinsonian medication. Movements of flexion and extension were applied through a 53° angle, at a constant frequency of 0.5Hz. Torque and angular displacements were measured by sensor. Twenty cycles of flexion and extension were executed.	Significant differences in rigidity were found in: (a) basal activation between PD patients and healthy controls; (b) in “on” state vs. “off” states; (c) in contralateral activation vs. passive assessment.
Sepehri, 2007 [51]	Case–control	52 people with PD	Servomotor with a transducer system and a potentiometer.	Elbow	An examiner was trained to execute flexion and extension movements at a constant speed (1 cycle/sec). In some cases, EMG was used to check the absence of voluntary muscle activity.	There was no correlation between age and severity of rigidity. Normalized total hysteresis had the greatest correlation for rigidity. This means that the measure of viscous properties may better score the level of the disease than the elastic ones.
Huang, 2016 [52]	Case–control	21 people with PD 14 people with stroke 22 healthy controls	Servomotor with electric goniometer.	Elbow	Two initial positions were measured, at 50° and 130° of flexion. Six pendulum tests were executed to register the number of oscillations, number of peaks and troughs during the swing, and	Significant differences in the number of oscillations were found between PD vs. controls. There were no differences between PD and stroke patients. Differences in relaxation index were found between controls vs. PD and
					relaxation index.	stroke. Differences in stiffness coefficient and damping coefficient were found between the two initial positions (greater in extension).
Nuyens, 2000 [53]	Case–control	10 people with PD 10 healthy controls	Isokinetic device with servomotor and electromyography.	Knee	Clinical evaluation was carried out by AS. Knee movements were executed at 60°, 180°, and 300°/s. Torque and EMG activity of quadriceps femoris, hamstrings, and gastrocnemius medialis muscles were recorded.	The greatest torque took place during flexion movements and high speeds. Rigidity depended on speed and direction of the movement, as well as coactivation of stretched and shortened muscles, especially during extension movements.
Anastasopoulos, 2009 [54]	Case–control	14 people with PD 23 healthy controls	Bárány chair with a cervical stabilizer.	Neck	Rotation of head and trunk. Stimuli were applied in darkness and without any auditory cues for the subject.	PD patients had more difficulty relaxing neck muscles than controls. PD showed greater peak torque than control.
Cano de la Cuerda, 2014 [55]	Case series	36 people with PD	Servomotor (Biodex System isokinetic dynamometer).	Trunk	A passive flexion movement was applied in a range of 80°, and an extension movement of 30° was applied at three velocities: 30°/s, 45°/s, and 60°/s.	The greatest resistance was obtained at the end of the range for both movements at every speed. There was a correlation between extensor rigidity and clinical scales (H&Y and UPDRS) for all speeds. There was a correlation between Schwab and England scale and flexor and extensor rigidity.

Abbreviations: AS, Ashworth scale; CRS, clinical rigidity score; EMG, electromyography; H&Y, Hoehn and Yahr; ROM, range of motion; UPDRS, unified Parkinson’s disease rating scale.

**Table 5:**
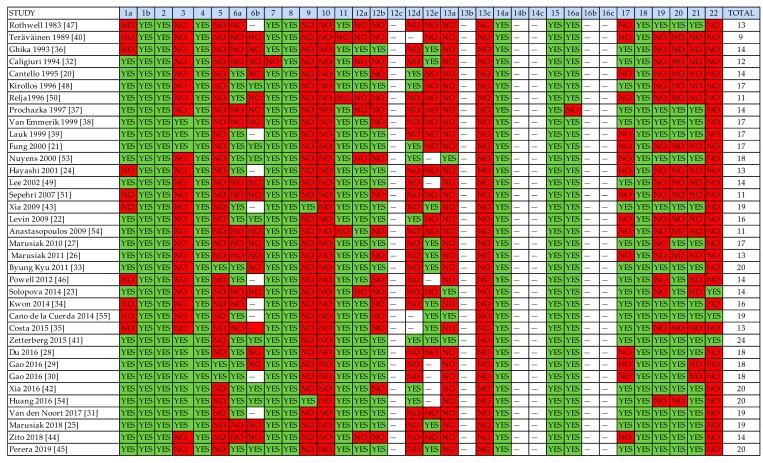
Strengthening the Reporting of OBservational Studies in Epidemiology (STROBE) scores of included studies.
